# A computational framework for qualitative simulation of nonlinear dynamical models of gene-regulatory networks

**DOI:** 10.1186/1471-2105-10-S12-S14

**Published:** 2009-10-15

**Authors:** Liliana Ironi, Luigi Panzeri

**Affiliations:** 1 IMATI-CNR, via Ferrata 1, 27100 Pavia, Italy

## Abstract

**Background:**

Due to the huge amount of information at genomic level made recently available by high-throughput experimental technologies, networks of regulatory interactions between genes and gene products, the so-called *gene-regulatory networks*, can be uncovered. Most networks of interest are quite intricate because of both the high dimension of interacting elements and the complexity of the kinds of interactions between them. Then, mathematical and computational modeling frameworks are a must to predict the network behavior in response to environmental stimuli. A specific class of Ordinary Differential Equations (ODE) has shown to be adequate to describe the essential features of the dynamics of gene-regulatory networks. But, deriving quantitative predictions of the network dynamics through the numerical simulation of such models is mostly impracticable as they are currently characterized by incomplete knowledge of biochemical reactions underlying regulatory interactions, and of numeric values of kinetic parameters.

**Results:**

This paper presents a computational framework for qualitative simulation of a class of ODE models, based on the assumption that gene regulation is threshold-dependent, i.e. only effective above or below a certain threshold. The simulation algorithm we propose assumes that threshold-dependent regulation mechanisms are modeled by continuous steep sigmoid functions, unlike other simulation tools that considerably simplifies the problem by approximating threshold-regulated response functions by step functions discontinuous in the thresholds. The algorithm results from the interplay between methods to deal with incomplete knowledge and to study phenomena that occur at different time-scales.

**Conclusion:**

The work herein presented establishes the computational groundwork for a *sound *and a *complete *algorithm capable to capture the dynamical properties that depend only on the network structure and are invariant for ranges of values of kinetic parameters. At the current state of knowledge, the exploitation of such a tool is rather appropriate and useful to understand how specific activity patterns derive from given network structures, and what different types of dynamical behaviors are possible.

## Background

Although, up to now, there is no model that efficiently and accurately represents the gene interactions underlying regulatory mechanisms in their whole complexity, recent experimental evidence seems to confirm the adequacy of a specific class of ODEs to describe their essential dynamical features. These models assume that genes are controlled by transcriptor factors, and that the effect of a transcription factor on the transcription rate of a gene, *the response function*, is threshold-dependent. Such switch-like behaviors across variable thresholds are mathematically well represented by steep sigmoidal functions.

Although these models provide detailed description of gene regulatory molecular mechanisms [1-3], their predictive usefulness, at quantitative level, is rather limited even when the network at hand is very well studied. In fact, the exploitation of classical numerical approaches is mostly impracticable as precise and quantitative information on (i) the biochemical reaction mechanisms underlying regulatory interactions, and (ii) kinetic parameters and threshold concentrations are currently unknown and not identifiable from available data. However, at the current state of knowledge, qualitative predictions of the dynamical properties are not make-shift solutions but rather appropriate to get insight into the functioning of systems not completely understood as molecular interaction networks are.

The application of generic qualitative simulation approaches, as originally proposed in the Artificial Intelligence research framework [4] under the label QR, is not the proper solution. The mathematical tools they are grounded on are too simple to compensate for knowledge incompleteness. This results in a number of drawbacks, e.g. their inability to upscalability, the exponential growth of the generated behaviors, and the generation of spurious behaviors, that seriously limit the range of applicability of such methods to predict the nonlinear dynamics of regulatory networks. A qualitative study of GRNs dynamics could, in theory, be performed by analytical methods [5-8] based on the classical theory of qualitative analysis of dynamical systems, and properly adapted to the specific class of models. But, in practice, the network complexity makes quite hard or even unfeasible traditional analysis.

Pioneering work towards automated qualitative analysis and simulation of GRNs has resulted in a computational tool, called GNA[9]. Although GNA has been successfully applied to study real world networks, namely the initiation of sporulation in *Bacillus subtilis *[10], and the response to nutritional stress and carbon starvation in *Escherichia coli *[11,12], it is not flawless. GNA circumvents the hard problem of dealing with sigmoidal nonlinear response functions by approximating them with step functions, discontinuous in the threshold hyperplanes. Such an assumption considerably simplifies the analysis as the model results in piecewise-linear equations, but it raises the problem to find a proper continuous solution across the threshold hyperplanes, or, in other words, to seek for generalized solutions of ODEs with discontinuous right-side terms. The solution to this problem is not straightforward as (i) there exist in the literature several definitions of generalized solutions, (ii) it is not yet completely understood what are the relationships between different definitions, and then, (iii) it is not clear how to choose the "right" definition for a particular task [13]. GNA adopts the Filippov approach [14] that results particularly convenient to deal with control problems but it may present drawbacks when applied to approximate the limit solutions of a continuous ODE model: it might find "too many" solutions, and fail to reach all stable ones. Thus, GNA suffers from the same problems, that in addition to those raised by a further approximation introduced to deal with computational issues, might compromise its soundness and completeness (Dordan O, Ironi L, Panzeri L, Some critical remarks on GNA, *in preparation*).

The qualitative simulation algorithm we propose works under the assumptions that (i) threshold-dependent regulation mechanisms are modeled by continuous steep sigmoid functions, and (ii) any two genes are never regulated at the same threshold by a certain variable. The sigmoidal-nonlinearities make the problem quite hard to be tackled. But, the assumption that all sigmoids have very high steepness allows us to apply a systematic way of analysis. Let us observe that, due to the switch-like behavior of the response functions around the thresholds, the GRN dynamics occurs at different time-scales. To be able to deal with both slow and fast nonlinear dynamics we theoretically base our algorithm on a classical singular perturbation analysis method properly adapted to the assumed class of ODEs [8,15]. Such a method suitably combined with QR key concepts computationally drives, starting from initial conditions, the construction of all possible state transitions, and calculates the sets of symbolic inequalities on parameter values that hold when specific transitions occur.

### A class of models of GRN dynamics

**Figure 1:**
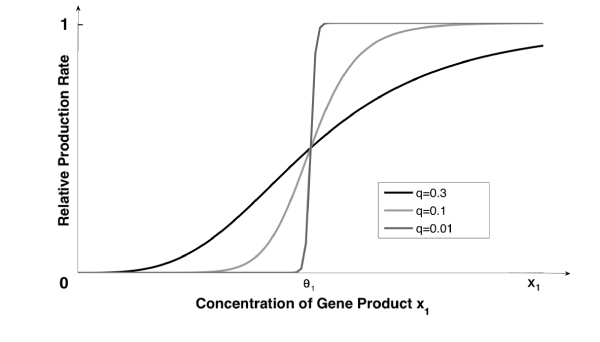
**Response function shape**. The sigmoid response function *S*(*x*_1_, *θ*_1_, *q*) describes the relationship between the concentration of a gene product *x*_1 _and the relative production rate of the regulated gene. The parameter *q *determines its steepness around the threshold *θ*_1_.

As phenomenological model of the complex dynamics of GRNs made up of *n *components, we consider the following generic equations:

(1)

where the dot denotes time derivative, *x*_*i *_is the concentration of the *i*-th gene product, *γ*_*i *_> 0 is the decay rate of *x*_*i*_, **Z **is a vector with *Z*_*jk *_as components, and *Z*_*jk *_= *S*(*x*_*j*_, *θ*_*jk*_, *q*) is a sigmoid function with threshold *θ*_*jk*_. The response, or regulatory, function *S *: R^+ ^→ [0, 1] is a continuous monotonic S-shaped map depending on the parameter *q *(0 <*q *≪ 1), that determines the steepness of *S *around the threshold value *θ*_*jk*_, such that for *q *→ 0 we have *S*(*x*_*j*_, *θ*_*jk*_, *q*) = 0 (respectively 1) when the value of *x*_*j *_is smaller (greater) than *θ*_*jk *_(Fig. 1).

Each *x*_*i*_, with domain Ω_*i *_⊂ R^+^, is associated with *n*_*i *_thresholds ordered according to *θ*_*ij *_<*θ*_*ik *_if *j *<*k*. The state equations describe the balance between the production process *f*_*i*_(**Z**) and the degradation one, herein supposed to be linear. The functions *f*_*i *_are multilinear polynomials in the variables *Z*_*jk*_, and are frequently composed by algebraic equivalents of Boolean functions. More precisely,

(2)

where *κ*_*il *_are real values that denote kinetic rate parameters, *L*_*i *_is the possibly empty number of interactions that synthesize *x*_*i*_, and, in accordance with the network structure, *α*_*jkl *_assumes value either equal to 1 when *Z*_*jk *_takes part in the *l*-th interaction or equal to 0 otherwise.

The validity of the biological assumptions underlying the model described by Eq. (1) has been confirmed by recent experimental evidence [16-28] and theoretical studies [29-32]. Thus, we can reasonably assume that such a model is suitable to give a phenomenological description of a wide range of regulatory systems in which the combined effects of a series of genetic processes, e.g. transcriptional and translational regulation, protein-protein interactions, metabolic processes, etc., can be properly described by threshold-dependent response functions.

**Figure 2:**
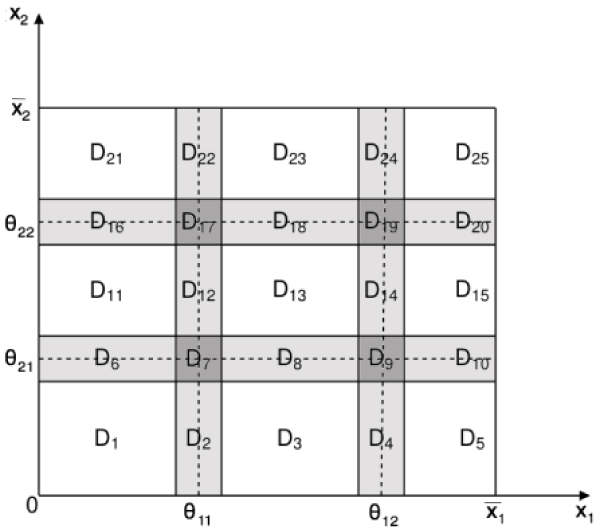
**An example of phase space partition**. Partition of the phase space associated to a dynamical system with two state variables, where each variable *x*_*i *_∈ [0, ], *i *= 1, 2 is associated with two ordered thresholds *θ*_*ij*_, *j *= 1, 2. The white rectangles denote regular domains. The gray rectangles denote switching domains, whose width, *δ *> 0 is a monotonic function of the parameter *q *with *δ*(*q*) → 0 for *q *→ 0. In the light gray regions one variable is switching and the other one is regular, whereas in the dark gray ones both variables are switching.

Let us represent the dynamics of the *n *state variables *x*_*i*_, modeled by the Eq. (1) and associated with appropriate initial conditions, in the phase space. The ordered sets Θ_*i *_of the *n*_*i *_threshold values *θ*_*ij *_associated with each *x*_*i *_naturally induce a partition of the phase space into qualitatively distinct domains. In the set Δ of all the domains identified by the partition, we can distinguish the set Δ_*s *_⊂ Δ of *switching domains *from the set Δ_*r *_⊂ Δ of *regular domains*, such that Δ = Δ_*s *_∪ Δ_*r *_(Fig. 2).

A regular domain *D*_*r *_(also called a *box*) is an open rectangular region between adjacent threshold hyperplanes in which the values of all response functions *Z*_*jk *_equal either 0 or 1. A switching domain *D*_*s *_is a narrow region, whose width depends on the parameter *q*, surrounding either a section of a threshold hyperplane or an intersection of threshold hyperplanes. In a switching domain *D*_*s *_we distinguish *σ *(*D*_*s*_) switching variables, *x*_*s*_, from *n *- *σ *(*D*_*s*_) regular ones *x*_*r*_. The values of a variables *x*_*s *_lies in the neighborhood of one of its thresholds and *Z*_*sk *_takes values in (0,1), while the values of a variable *x*_*r *_lies between two adjacent thresholds. The network dynamics in each domain *D *∈ Δ is described by different models. The motion in *D*_*r *_∈ Δ_*r *_is described by linear ODEs, and its analysis is straightforward. The motion in *D*_*s *_∈ Δ_*s*_, or equivalently around the thresholds, is described by nonlinear equations, and occurs at different time-scales. Therefore, to capture the salient features of the nonlinear dynamics in a switching domain, and to determine how the trajectories cross it to move towards other domains, a specific mathematical method is required.

## Methods

### Singular perturbation analysis in the switching domains

Singular Perturbation Analysis (SPA) is a classical approach to study phenomena that occur at different time-scales [33]. The dynamics of such phenomena are described by ODEs, associated with appropriate initial conditions, in which a small parameter (0 <*q *≪ 1) multiplies either one of the derivatives or higher order derivative. Let us indicate this initial value problem by ℳ_*q*_, and by ℳ_0 _the same problem where *q *= 0. As *q *→ 0, the solution of ℳ_*q *_identifies a "small" region, called *boundary*-*layer region*, of non-uniform convergence to the solution of the *reduced system *ℳ_0_. The region of uniform-convergence of ℳ_*q *_to ℳ_0 _is called *outer region*. Taken together, the outer and boundary-layer solution approximate the solution of ℳ_*q *_for small nonzero values of *q*.

Such a general approach is not directly applicable to our problem, but it must be tailored to it [8]. In outline, (i) the Eqs. (1) related to the *x*_*s *_variables are rewritten into the standard form of SPA through a change of coordinate system, (ii) the boundary-layer and outer solutions are calculated in the new coordinates, and (iii) they are converted back into the usual frame of reference.

Let Σ: Ω ↦ [0, 1]^*n *^be the coordinate transformation that converts the *x*_*s *_coordinates into the *Z*_*s *_ones. As under our assumptions, , where *d*_*s *_is a continuous and limited function, we can rewrite Eq. (1) as in the following:

(3)

where **Z**_**S**_, **Z**_**R **_are the vectors of switching and regular variables *Z*_*s *_and *Z*_*r*_, respectively.

The fast dynamics in the boundary-layer is studied by suitably scaling the time variable, namely *τ *= *t*/*q*. In the limit, the fast dynamics is obtained by solving the system:

(4)

associated with appropriate initial conditions, where the prime denotes the derivative with respect to *τ*. As **Z_R _**is constant in any *D*_*s *_∈ Δ_*s*_, we focus on the switching variables *Z*_*s *_only. The system (4) has a manifold of stationary points, called slow-manifold, given by the solutions, for all *s*, of the stationary equations  = 0. We call *exit point set *(*EP*) the set of points in the slow-manifold that are stable and satisfy the Tikhonov-Levinson theorem, and we call *Z*-*cube Ƶ*(*D*_*s*_) =  the frame of reference where we search for exit points.

The motion in the original time *t *along the exit points is described by the reduced system ℳ_0_. Thus, under the hypothesis that at least one exit point  exists, the motion equations of regular variables, or equivalently the outer solution, is represented by:

(5)

The problem (5) is linear, and then, given the initial conditions, the outer solution, that determines how the trajectories move along the *x*_*r *_directions, is easily calculated.

The calculation of the slow-manifold generally leads to an heavy, nonlinear computational problem as it consists in the solution of a set of polynomial equations. In the current implementation we adopt a further assumption that sounds realistic:

Assumption . Every gene product only regulates one gene at each of its thresholds

Such an assumption considerably simplifies the calculation of the slow-manifold. Mathematically, it implies that each *Z*_*jk *_only occurs in one equation, and thus the terms *f*_*s*_(**Z**_**R**_, **Z**_**S**_) are linear.

Remark 1

The location of each exit point is crucial in our analysis as it indicates the next adjacent domains the trajectories are moving towards along the *x*_*s *_directions.

Let us observe that stationary points always exist on the vertices of *Ƶ*(*D*_*s*_) as they are the roots of *d*_*s*_(*Z*_*s*_, *θ*_*s*_) = 0. The computational cost of the search for all the other exit points could be quite high, but it can be considerably reduced by checking first a necessary condition for the existence of a stationary point on the other elements of *Ƶ*(*D*_*s*_). Let *F *be a face or the interior of *Ƶ*(*D*_*s*_). In [15], it has been proved that necessary condition for the existence of a stationary point in *F *is that the Jacobian matrix  restricted to the switching variables in *F *has a complete loop. This holds if and only if there is a non-zero loop involving all variables in **J**_*F*_, and can be checked by using concepts from graph theory.

Let  be a stationary point located on  ∈ *Ƶ*(*D*_*s*_), and  = {*l *: *l *∈ {l,..., *σ *(*D*_*s*_)},  ∈ {0, 1}}.  is an exit point if (i) it is stable. If  is on the boundary of *Ƶ*(*D*_*s*_), it is required, in addition to (i), that (ii) *Z*_*l*_, ∀ *l *∈ , heads towards . The stability of  is checked by analyzing the spectrum of the Jacobian matrix, and the condition (ii) is verified when, ∀ *l *∈ , the sign of , in a neighborhood of  is in agreement with the motion of *Z*_*l *_towards . More precisely, in a neighborhood of ,  > 0 and  = 1 or  < 0 and  = 0.

Remark 2

SPA works out in the limit *q *→ 0, but the calculated solution approximates the solution of Eq. (3) for sufficiently small *q *(0 <*q *≪ 1). Moreover, it can be proved that the Jacobian matrix is stable for 0 <*q *< ≪ 1. Thus, the exit points calculated in the limit are also valid for values of *q *<.

Remark 3

Under the *Assumption *, the reduced equations are always independent of the variables *Z*_*s *_in the Eq. (4), and the two sets of equations are mutually independent. Thus, the behavior of the variables *x*_*s *_in a Z-cube is completely independent of the values of variables *x*_*r*_, and the motion in a switching domain may be studied by first analyzing the former variables, and then the latter ones.

### Qualitative reasoning concepts

Among the generic qualitative approaches proposed in the literature, QSIM provides both the most suitable formalism and algorithm to represent and simulate models qualitatively abstracted from ODEs [4]. Thus, we revise and *ad hoc *tailor its key concepts to our specific class of models.

#### Qualitative value

The real values of each state variable *x*_*i *_with domain Ω_*i *_= [0, ] are discretized into a finite ordered set of values qualitatively important from the biological point of view. In our context, the *qualitative value space *of each *x*_*i *_is defined by the ordered set Θ_*i *_made up of both its *n*_*i *_threshold symbolic values and the endpoints of Ω_*i*_. The partition of the whole system domain, induced by the sets θ_*i*_, *i *= 1,..., *n *identifies qualitatively distinct hyper-rectangles *D *that define all possible *system qualitative values*.

#### Qualitative state

Let *A*(*D*) be the set of domains adjacent to *D *∈ Δ. The *qualitative state *of *D*, *QS*(*D*), is defined by all of its adjacent domains *D*_*k *_towards which a transition from it is possible:



Each transition from *D *identifies a domain next traversed by a system trajectory. More precisely, if we number by *i *the domain *D *traversed at time *t*_*i*_, each *D*_*k *_∈ *QS*(*D*) will be traversed by different trajectories at time *t*_*i*+1_.

#### State transition

Qualitative simulation of network dynamics is achieved by iteratively applying local transition strategies from one domain to its adjacent domains. The possible transitions from any *D *are determined by different strategies according to whether *D *∈ Δ_*r *_or *D *∈ Δ_*s*_.

In the case *D *∈ Δ_*r*_, like in traditional QR methods and in GNA[9], possible transitions are determined by the signs of . As  are defined by linear expressions, such signs are easily determined by exploiting the inequalities that define the parameter space domain.

In the case *D *∈ Δ_*s*_, a sign-based strategy is not practicable as the expressions for  are nonlinear. A convenient way to proceed is given by SPA: transitions from *D *towards adjacent *D*_*k *_are determined by the locations of the exit points on the boundary elements of the associated *Ƶ*(*D*). Due to *Assumption *, each boundary element of *Ƶ*(*D*) may contain at most one exit point. But, as, in a qualitative context, knowledge incompleteness on the parameter values is expressed by coarse constraints, the boundary element of *Ƶ*(*D*) where an exit point is located is not, in general, uniquely determined. Thus, unless the exit point is located in the interior of *Ƶ*(*D*) and a stable solution is reached, the successors of *D *are not uniquely determined. However, through symbolic computation procedures, it is possible to calculate the set of inequalities, , on parameter values that hold when the transition from *D*_*i *_to *D*_*k *_occurs. Then, the 3-tuple ⟨*D*_*i*_, *D*_*k*_, ⟩ clearly and uniquely identifies each path from *D*_*i *_to *D*_*k*_.

#### Qualitative behavior

A finite sequence of paths, where each path is clearly both linked and consistent with its predecessor and successor, defines a *qualitative behavior*: , where *D*_0 _is the initial domain, and *D*_*F *_either contains a stable fixed point or identifies a cycle, i.e it is an already visited domain. *I*_0 _is the initial set of inequalities that defines the parameter space domain, and *I*_*F *_the set of inequalities on parameter values associated with *D*_*F*_.

#### Qualitative simulation

Starting from an initial domain *D*_0 _and a set, *I*_0_, of symbolic inequalities on parameter values, qualitative simulation generates all possible state transitions, and represents them by a directed tree rooted in *D*_0_, BT(*D*_0_), where the vertices correspond to *D*_*i*_, and the arcs, labeled by the inequalities , to the transitions from *D*_*i *_to *D*_*j*_. Each branch in BT(*D*_0_) defines a qualitative trajectory *QB *from *D*_0_, that traverses specific domains and occurs when the values of parameters satisfy its related inequalities. Such a trajectory abstracts all those numerical solution of the ODE model with initial conditions **x**^0 ^varying in *D*_0_, and with kinetic parameter values fulfilling the inequalities.

## Results

Given as input, (i) *n *symbolic state equations of the form (1); (ii) *n *qualitative value spaces Θ_*i *_= {*θ*_*ij*_} of the state variables; (iii) *D*_0 _∈ Δ; (iv) a set of symbolic inequalities *I*_0 _on parameters defining a parameter space domain *PSD*_0_, the algorithm, through an iterative procedure initialized by ⟨*D*_0_, *I*_0_⟩, provides as output the entire range of possible network dynamics represented by BT(*D*_0_). Its main steps are outlined in the following:

1. *Define *the qualitative values by partitioning the phase space into regular and switching domains.

2. *Calculate *the qualitative state *QS*(*D*_*i*_) of the current domain *D*_*i*_.

(a) Calculate the *symbolic state equations in D*_*i*_, and the *set A*(*D*_*i*_);

(b) Determine *constraints **on parameters *that need to be fulfilled to have a transition from *D*_*i *_to *D*_*k *_∈ *A*(*D*_*i*_);

(c) Check *consistency of **with I*_0_, and build the path *e*_*ik *_= *D*_*i *_→ *D*_*k *_;

3. *Append ⟨D*_*i*_, *D*_*k*_, ⟩ to BT(*D*_0_), and *mark D*_*i *_as visited domain.

4. *Repeat *from step 2 for each not visited *D*_*k*_.

Step 2, and in particular the calculation of the conditions on parameters , is the core of the overall algorithm. A transition from *D*_*i *_to *D*_*k *_actually occurs, and then *D*_*k *_∈ *QS*(*D*_*i*_), only if the set  is consistent with the set *I*_0_, i.e. when it defines a not empty parameter space domain  such that  ⊆ *PSD*_0_. The calculation of an inequality set  is performed by two distinct algorithms that implement a different transition strategy according to whether *D*_*i *_∈ Δ *r *or *D*_*i *_∈ Δ_*s*_.

### Moving from a regular domain

The algorithm in charge of the construction of the possible paths from regular domains is, in principle, similar to that one proposed by GNA, but it is more informative as it calculates the s. In the limit, we indicate *D*_*i *_∈ Δ_*r *_by the product  where (*θ*_*ji*_, *θ*_*j*(*i*+1)_) denotes the interval of *x*_*j *_in *D*_*i*_.

(a) *Symbolic state equations in D*_*i *_∈ Δ_*r*_. In each box *D*_*i*_, *Z*_*jk *_equals either 0 or 1 in the step function limit. This simplifies Eq. (1) as they reduces to linear equations:

(6)

where *μ*_*j *_depends on *D*_*i*_, and is given by the sum of some *κ*_*jl*_s. From Eq. (6) we can easily find the *focal point * the trajectories are heading towards. Herein, we assume that focal points do not belong to switching domains. If x* belongs to *D*_*i*_, there is a stable point in it. Such a stable point exists in *D*_*i*_, i.e. *D*_*i *_∈ *QS*(*D*_*i*_), if the set of inequalities  ∀*j *∈ {1,..., *n*} exists and is consistent with *I*_0_. Otherwise, the trajectories move towards a switching domain *D*_*k *_adjacent to *D*_*i*_.

(b) *Constraints **on parameters*. ∀ *D*_*k *_∈ *A*(*D*_*i*_), the algorithm calculates the set of inequalities on parameters  that need to be fulfilled to have a transition from *D*_*i *_to *D*_*k*_. As in *D*_*i *_all the equations (6) are linear, and all the trajectories head towards a focal point x* in a regular domain, such inequalities are calculated by imposing that the signs of state variable rates match the relative position of *D*_*k *_with respect to *D*_*i*_. We define the relative position of *D*_*k *_with respect to *D*_*i*_, indicated by  where *v*_*j *_∈ {-1, 0, 1}, through the comparison of the intervals defining *D*_*i *_and *D*_*k*_. , initialized to *I*_0_, is updated, ∀ *j *∈ {1,..., *n*}, with either the inequality  if *v*_*j *_= 1 or  if *v*_*j *_= -1.

(c) *Possible transitions from D*_*i *_*to D*_*k*_. If the calculated inequality set defines a not empty parameter space domain  ⊆ *PSD*_0 _then a transition towards *D*_*k *_is possible and the qualitative state *QS*(*D*_*i*_) is updated accordingly.

To exemplify how the algorithm works we consider the ODE model:

(7)

where the parameters are positive, and all the response functions are expressed by the Hill function commonly used in the literature.

Let us consider the domain *D*_11 _in Fig. 2 as current *D*_*i*_, and let us define *I*_0 _as follows:

(8)

The conditions on parameters in Table 1 that are consistent with *I*_0 _identify the possible transitions from *D*_11 _towards adjacent domains in *A*(*D*_11_) = {D_6_, *D*_7_, *D*_12_, *D*_16_, *D*_17_}. Among the inequalities in Table 1,  is the only one in agreement with *I*_0_. Thus, *QS*(*D*_11_) = {*D*_12_} and the only possible transition from *D*_11 _occurs when  ∧ *I*_0 _holds.

**Table 1:** Example parameter inequalities

Inequality Constraints		
	*Sign Constraints*	*Parameter Inequalities*
	< 0	
	> 0	
	> 0, < 0	
	> 0	
	> 0, > 0	

### Moving from a switching domain

In a domain *D*_*i *_∈ Δ_*s*_, the nonlinear dynamics is characterized by fast and slow motions, respectively associated with *x*_*s *_and *x*_*r*_, that are independently calculated. Let us reindex the variables *x*_*j*_, *Z*_*j *_such that the switching variables come first, and proceed first with the construction of the fast motion by exploiting the SPA approach.

#### A – Fast motion

The study of the fast dynamics is performed in *Ƶ*(*D*_*i*_) in the scaled time *τ *= *t/q*, and aims at localizing the set of exit points in *Ƶ*(*D*_*i*_) rather than at detailing the dynamics within it. Such points clearly identify the next adjacent domains the trajectories are moving towards from *D*_*i *_along the *x*_*s *_directions.

To this end, the algorithm defines, in the limit, the mapping :  → *Ƶ*(*D*_*i*_), where  = *D*_*i *_∪ *A*(*D*_*i*_), such that the interior of *Ƶ*(*D*_*i*_), and its boundary are the images of *D*_*i*_, and *A*(*D*_*i*_), respectively. More precisely, the domains *D*_*k *_∈ *A*(*D*_*i*_) are mapped into the faces of *Ƶ*(*D*_*i*_) when *D*_*k *_∈ Δ_*s *_or into its vertices, otherwise. Let ℱ be the set of both the faces and the interior of *Ƶ*(*D*_*i*_), and *F *its generic element.

**Figure 3:**
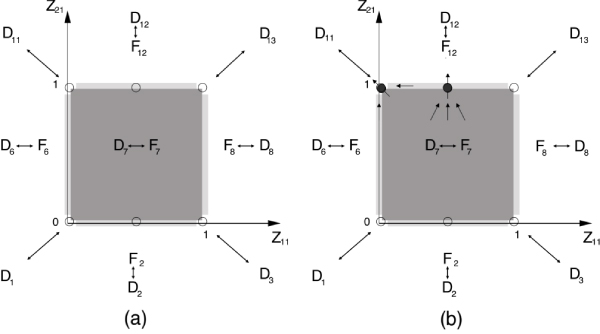
**Mapping between *D*_*i *_and *Ƶ*(*D*_*i*_)**. (a) Mapping of *D*_7 _and its adjacent domains in Fig. 2 into the elements of *Ƶ*(*D*_7_). The empty circles on the boundary of *Ƶ*(*D*_7_) denote the candidate exit points. (b) The filled circles denote the exit points from *Ƶ*(*D*_7_), whereas the empty circles denote unstable stationary points that correspond to possible entrance points to *Ƶ*(*D*_7_). The single-headed arrows denote the direction of change of *Z*_*l*_, ∀*l *∈ ℒ_*F *_in a neighborhood of the exit points.

To exemplify, let us consider the switching domain *D*_7 _in Fig. 2. The set ℱ has five elements, the four faces corresponding to *D*_2_, *D*_6_, *D*_8_, *D*_12_, and the interior of *Ƶ*(*D*_7_) that correspond to *D*_7_. Moreover, the vertices of *Ƶ*(*D*_7_) are the images, through the mapping Σ, of the adjacent regular domains *D*_1_, *D*_11_, *D*_3_, and *D*_13 _(Fig. 3).

#### (a) 1. Symbolic state equations in D_*i *_∈ Δ_*s*_

The algorithm symbolically calculates the boundary-layer equations (4) in the *Z *variables associated with the current switching domain.

As an example, let us consider the model (7), and the domain *D*_7_, where both variables *x*_1_, *x*_2 _are switching in it, respectively, around the thresholds *θ*_11_, *θ*_21_. In *D*_7_, characterized by fast motion only, the boundary-layer system is given by:

(9)

#### 2. Identification of the candidate next traversed domains D_*k*_

Among all the domains *D*_*k *_∈ *A*(*D*_*i*_) only those that correspond to an element on the boundary of *Ƶ*(*D*_*i*_) where a stationary point is located are possible domains next traversed by the trajectories. Then, the algorithm first builds the subset of elements of *A*(*D*_*i*_) that are candidate successors of *D*_*i*_.

Let us denote by *EP *the set of stationary points, initially made up of the vertices of *Ƶ*(*D*_*i*_). Under the *Assumption A*, each element *F *∈ ℱ, may contain at most one stationary point. A necessary condition for the existence of a stationary point on *F *is the presence of a non-zero loop in **J**_*F*_, the Jacobian matrix calculated on *F*. Then, ∀ *F *∈ ℱ, the algorithm calculates **J**_*F *_and searches for a non-zero loop involving all variables in **J**_*F*_. In case, it symbolically calculates the stationary point on F, and updates accordingly the set of candidate exit points *EP*. Let us observe that one internal stationary point exists if all variables in *Ƶ*(*D*_*i*_) are involved in a loop.

In the example, only  and  have a non-zero loop. Then, the algorithm looks for the stationary state on *F*_2 _and *F*_12_:  and . Finally, the exit point candidate set is updated with the points  and  (Fig. 3(a)).

#### (b) Constraints  on parameters

The inequality set , initialized to *I*_0_, is calculated for each candidate exit point  by requiring that each point is stable and fulfills other motion conditions on parameters. More precisely, the algorithm:

1. analyzes the spectrum of the Jacobian matrix **J**_*F*_, and imposes possible conditions  on parameters to ensure stability of ;

2. imposes conditions *I*_*k*, *l *_on the sign of , ∀ *l *∈ ℒ_*F*_, in a neighborhood of  so that the motion of *Z*_*l *_heads towards ;

3. for those  located on elements of ℱ, imposes the further condition, *I*_*k*,(0, 1)_, 0 < < 1 for each  that does not take value 0 or 1.

Finally,  is an exit point if  holds.

Conditions 2. and 3. are easily checked, while the stability condition is checked by using concepts from graph theory, and the usual definition of stability based on the sign of the eigenvalues of **J**_*F*_. It follows from *Assumption * that the matrix **J**_*F *_is block-structured, where each block is a permutation matrix associated with a sub-loop. It can be proved that  is stable if: (i)  has no blocks with dimension strictly greater than 2; (ii) in 1-dimensional blocks the elements are negative; (iii) in 2-dimensional blocks the loop products are positive.

Remark 4

Let us consider the general case when *D*_*i *_∈ Δ_*s *_is characterized by both switching and regular variables. The motion from an exit point located in the interior of *D*_*i *_occurs in a sliding mode along a stable point in the slow-manifold of the boundary-layer system, and is described, in the normal time, by the reduced system. Then, a stable stationary point exists in *D*_*i*_, i.e. *D*_*i *_∈ *QS*(*D*_*i*_), if a stable point exists in the interior of *Ƶ*(*D*_*i*_), and the regular variable rates are *zero *in a point inside *D*_*i*_. The set of inequalities that checks the latter condition are defined by  ∀*j *∈ {*σ *(*D*_*i*_) + 1,..., *n*}.

#### (c) Possible transition from *D*_*i *_to *D*_*k*_

The exit points located on *Ƶ*(*D*_*i*_) clearly identify the set of all possible *exit domains*, i.e. those domains towards which a transition from *D*_*i *_is possible. Such domains are easily calculated by applying the map Σ^-1 ^to each element of *Ƶ*(*D*_*i*_) that contains an exit point. Let us observe that the remaining unstable stationary points in *EP *are possible entrance points to *D*_*i*_.

In the example, both  and  are 1-dimensional block matrices with negative elements. Then, both exit points  and  fulfill the stability condition 1. without further constraints imposed on the parameters. The condition 2. on variable *Z*_*l*_, *l *= 2 imposes:

(10)

(11)

The inequality *I*_12,2 _defined by (11) is compatible with (8), but the inequality *I*_2,2 _is not. Then,  is removed from the exit point set. To be an exit point  must satisfy the condition 3.:



Finally,  is an exit point if , defined by *I*_0 _∧ *I*_12,2 _∧ *I*_12,(0,1)_, holds.

As for vertices in the example, the conditions 1. and 2. are fulfilled in the point  = (0, 1) that corresponds to the vertex defined as image of *D*_11 _by the map Σ.

Finally, the only exit domains are *D*_12_, *D*_11_, and then *QS*(*D*_7_) = {*D*_12_, *D*_11_} (Fig. 3(b)).

#### B – Slow motion

The slow motion of regular variables *x*_*r *_along the exit points is studied in the normal time *t *in the usual frame of reference, and it is reconstructed from the reduced system (5) through the same symbolic procedure given for regular domains.

### Qualitative simulation outcomes

Through the described iterative procedure, the algorithm builds (i) a behavior tree, rooted in the initial domain, and (ii) sets of inequalities on parameter values that characterize specific state transitions. The behavior tree captures the entire spectrum of network dynamical behaviors that are invariant for ranges of parameter values defined by the calculated inequalities.

**Figure 4:**
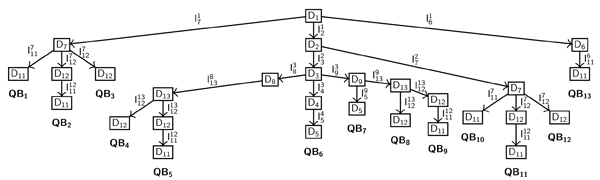
**Behavior tree**. Behavior tree rooted in *D*_1_, BT(*D*_1_), obtained by the simulation of the model Eqs.(7) with initial condition ⟨*D*_1_, *I*_0_⟩, where *I*_0 _is defined by Eq. (8). Each branch in the tree defines a qualitative trajectory that occurs when the inequalities that characterize each path in the branch hold.

**Figure 5:**
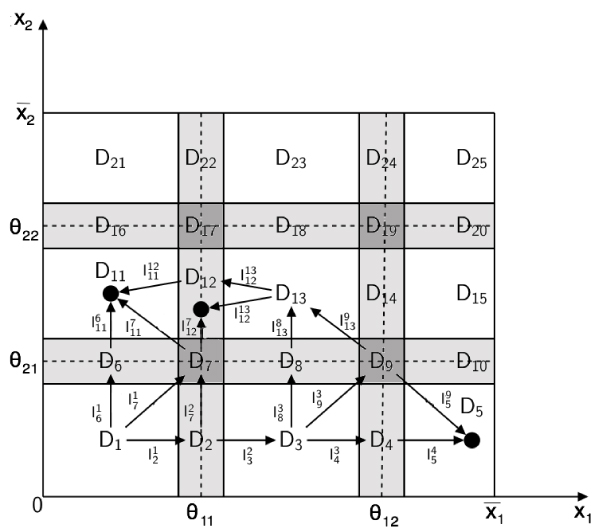
**Representation of the qualitative trajectories in the phase space**. Phase space representation of trajectories defined by BT(*D*_1_) in Fig. 4, filtered of the spurious behaviors *QB*_2 _and *QB*_11_. Stable states are denoted by ·. The labels  denote the sets of inequalities on parameter values that hold when transitions from *D*_*i *_to *D*_*k *_occur.

To illustrate the type of output the algorithm produces, let us consider the results of the simulation of the ODE model (7) starting from *D*_1 _with the parameter space defined by the inequalities *I*_0 _(8). Through the described iterative procedure, the algorithm builds the behavior tree, BT(*D*_1_), showed in Fig. 4, and calculates the inequalities on the parameters, listed in Table 2, that are associated with each path in BT(*D*_1_). As in the example *n *= 2, the trajectories described by the tree are also easily summarized in the phase plane (Fig. 5). Three reachable stable states, located in *D*_11_, *D*_12 _and *D*_5_, are identified by the final leaf of each branch in BT. As *D*_12 _∈ Δ_*s*_, one of them is a singular stable point whereas the others are regular stable points. These stable states are reached by different predicted qualitative behaviors, each of them occurring under specific constraints on parameters. For example, the trajectory *QB*_13 _starting from *D*_1_, crossing *D*_6_, and reaching a stable point in *D*_11 _is allowed when the inequalities  and *I*_11_, consistent with *I*_0_, hold.

**Table 2:** Inequalities associated with BT(*D*_1_)

, *I*_12_	*I*_0 _∧
	*I*_0 _∧
	*I*_0 _∧
	*I*_0 _∧
	*I*_0 _∧
*I*_5_	*I*_0 _∧
	*I*_0_

**Figure 6:**
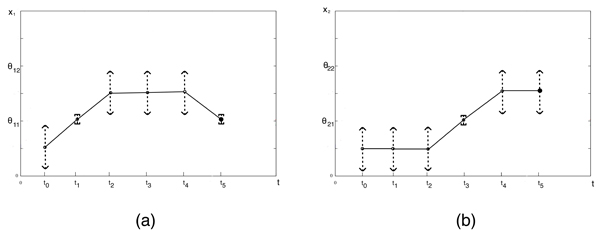
**Temporal representation of a qualitative trajectory**. Qualitative temporal representation of the behavior labeled *QB*_4 _in Fig. 4, namely of the variables *x*_1 _(a) and *x*_2 _(b). On the horizontal axis, the discrete sequence of symbolic instants at which the domains  are sequentially traversed. On the vertical axis, the qualitative value space of each variable is reported. The qualitative values (∘) abstract all real values in the related open and closed intervals, whose width is highlighed by a dashed line. The stable states are denoted by ·. The continuous line connecting the qualitative values has been introduced to give a hint of the qualitative profile.

**Figure 7:**
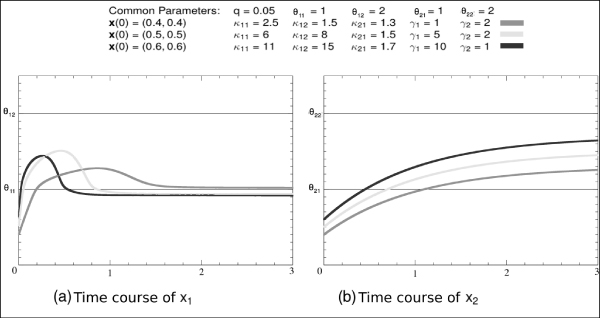
**Numerical simulation results**. Time courses of the variables *x*_1 _(a) and *x*_2 _(b) obtained by numerical simulations of the model (7). The different plots result from simulations with different initial numeric values in *D*_1_, and different sets of parameter numeric values but fulfilling the same inequalities that characterize the qualitative trajectory *QB*_4_. The horizontal continuous lines in correspondence to threshold values should facilitate the comparison with Fig. 6. All plots related to each variable are qualitatively abstracted by the related plot in Fig. 6.

In general, for *n *> 2, due to graphical difficulties, each branch in the tree is given a time representation. More precisely, a specific qualitative behavior, e.g. *QB*_4_, is described by the temporal evolution of each variable (Fig. 6). The behavior in Fig. 6 abstracts all numerical trajectories of the ODE model (7) obtained with different initial values in *D*_1_, and different sets of parameter values that fulfill the same inequalities associated with *QB*_4 _(Fig. 7).

## Discussion

The algorithm is currently under implementation. As for symbolic calculus, the implementation requires to tackle complex tasks, such as: (i) update an inequality set with an another one; (ii) check the consistency of two sets of inequalities *I*_1 _and *I*_2_; (iii) solve systems of equations; (iv) find cycles in the Jacobian matrix. As for (iii), the original equations are multilinear in *Z*_*s*_, but due to *Assumption * they can be straightforward solved and analyzed for stability. Also the solution of problems (i) and (ii) benefits from *Assumption * as the inequalities are always linear. Then, thanks to the *Assumption *, and to algorithms proposed both by the literature and common symbolic computation package, such as Mathematica [34], the tasks (i)-(iii) are simplified and feasible. As for the task (iv), it is performed by using cycle-detection algorithms and matrix graph theory tools [35].

### Soundness and completeness

#### Soundness

The algorithm guarantees that the behavior tree captures all of the sound behaviors for values of *q *sufficiently small. A closed-form expression of a symbolic upper bound of *q*, , in terms of model parameters guarantees the Jacobian matrix stability. Then, the generated behaviors are sound for values of *q *<. However, for a complete formal proof of soundness we need to prove that assuming, in the limit, stability instead of asymptotic stability does not affect the results for values of *q *<.

#### Completeness

At the current stage, the algorithm may generate spurious behaviors. There is a twofold explanation for that. First, we have not yet performed a thorough analysis with respect to entrance-exit transition, or in other words, we have not yet solved the problem of identifying the only admissible connections between entrance and exit points. Moreover, singular perturbation analysis is a *local *procedure that works quite well in a quantitative context but that needs, in a qualitative context, to be supported by a *global *criterion when local paths are connected to produce a specific trajectory. For example, the behavior *QB*_2 _is spurious as  is not consistent with . Similarly, *QB*_11 _is spurious.

The characterization of the paths from one domain to the next ones by sets of inequalities constraining the model parameters is quite new in the field of qualitative simulation, as for both general-purpose and specifically tailored algorithms. Such a strategy may reveal quite useful in the definition of a "global criterion" that allows us to distinguish sound behaviors from spurious ones by requiring that the sets of inequalities that label the local paths in a specific trajectory are consistent with each other. Both the definition of such a criterion and its implementation are not a trivial task, especially from a computational point of view. As for algorithm completeness, another essential methodological and computational issue to be deepened deals with the definition of the transition map that *properly *connects the entrance points to the exit points associated with a switching domain.

### Comparison between the algorithm and GNA

Until now, to the best of our knowledge, GNA is the only computational counterpart of the algorithm herein proposed. Unlike GNA, that simplifies the simulation problem by approximating the sigmoid response functions by step functions *discontinuous *in the thresholds, our qualitative simulation algorithm tackles the problem in all of its complexity, and works for models of GNAs with *continuous *sigmoid response functions. As a matter of fact, it has been designed to both provide a tool for a more realistic modeling framework and overcome the limits of GNA. Furthermore, in addition to more solid mathematical grounds for soundness and completeness, our algorithm differs from GNA as far as the required input information and the output outcomes are concerned. In GNA, the trajectories are constructed under the assumption that the locations of the focal points associated with the regular domains, symbolically expressed by a set of inequalities on parameters, are specified as input. But, as this precise knowledge is unlikely available, a thorough study of the dynamics of the system at hand might require several runs of the algorithm to simulate the GNA dynamics under possible different assumptions on focal point locations. Then, the study could result quite heavy due to the high number of possible scenarios, and the need to check, to avoid further causes of generation of spurious behaviors, the consistency of all the parameter inequalities that precisely localize a specific focal point.

**Figure 8:**
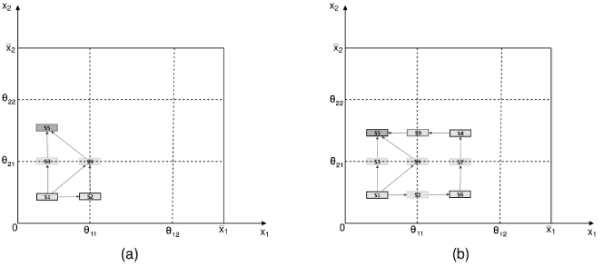
**GNA simulation outcomes**. Simulation outcomes of the example model obtained by the algorithm GNA given as input *D*_1 _and the parameter inequalities, compatible with *I*_0_, ; ; ;  (panel (a)), ; ; ;  (panel (b)). The states generated by GNA, and labeled *S*_*n*_, have been located at their position in the phase space to make the comparison with the results in Fig. 5 possible. Both sets of trajectories in (a) and (b) are subsets of the set of trajectories generated by our algorithm.

Our algorithm does not need precise information on the location of focal points but, to be fired, it just requires the parameter space domain, that is inequalities on parameters that univocally determine the sign of direction of change of each state variable in the initial domain. Afterwards, it is up to the algorithm to calculate and refine the parameter inequalities that distinguish each simulated behavior. Due to more relaxed constraints, a single run of our algorithm leads to a richer and more informative set of behaviors than that one obtained by GNA as it is highlighted by the comparison of Fig. 5 with Fig. 8. Let us observe that any other possible definition of parameter inequalities would not have led to the set of behaviors in Fig. 5 but to one of its subsets.

A complete and fair performance evaluation of our approach when compared with GNA would not disregard its application to those real world networks successfully simulated within the GNA framework, namely the initiation of sporulation in *Bacillus subtilis *[10], and the response to nutritional stress and carbon starvation in *Escherichia coli *[11,12].

### Application to Systems/Synthetic Biology

The qualitative simulation algorithm presented provides powerful computational grounds for *dry *experiments to be performed in both Systems and Synthetic Biology contexts. It is an economic tool for hypothesis testing and knowledge discovery as it allows us to understand how specific activation patterns are derived from models of fixed network structures, and which different dynamical behaviors are possible.

In a Systems Biology context, its exploitation is useful in the formulation phase of new hypotheses and theories that explain, in both physiological and pathological situations, experimental data either previously unobserved or not interpretable by the well-established biological knowledge. The result of the comparison of the simulated behaviors with the observed ones allows us either to confirm or to refuse the model that represents the new formulated biological hypotheses. Moreover, as qualitative simulation derives the entire range of network dynamics consistent with the initial conditions, the simulation outcomes might capture behaviors never observed. Such behaviors need to be experimentally tested by *ad hoc *designed experiments to possibly further confirm or suggest how to revise the model, i.e. the underlying biological hypotheses. The effective applicative potential of the proposed algorithm within a model-based reasoning cycle for the deep comprehension of complex real world biological systems is currently under study. More precisely, it will be applied (1) to explain and interpret the dynamics of the complex regulatory network that controls motility in *Bacillus subtilis *[36], and (2) to explore the molecular mechanisms that regulate the transition of a normal cell phenotype to an invasive-metastatic phenotype [37].

The model-based reasoning cycle results particularly convenient and useful also when applied to Synthetic Biology problems in the design phase. The construction of a synthetic biological network in the cell, able to perform specific tasks or to produce desired behaviors of a biological process in response to external stimuli, could benefit from a preliminary *dry *design of the network. To this end, models of different network structures can be simulated in correspondence to different inputs, i.e signals either exogenous or cellular, till the desired stable behaviors are obtained. Let us remind that, in our simulation framework, each specific simulated behavior is characterized by a chain of parameter inequalities. Thus, the actual construction of a synthetic network could benefit from such a piece of information as it provides the admissible parameter space domain for the target behaviors. The synthetic network IRMA[38] built in the yeast *Saccharomyces cerevisiae *is made up of a small number of components but rather complex in their interconnections as it includes multiple feedback loops generated by the combination of transcriptional activators and repressors. It offers a suitable benchmark for *in vivo *testing our computational framework.

## Conclusion

The assumption of continuous response functions makes the simulation problem hard to be tackled but it is crucial in view of the realization of tools that can be gradually extended to tackle more and more realistic models. Our algorithm is grounded on a set of symbolic computation algorithms that carry out the integration of qualitative reasoning techniques with singular analysis perturbation methods: the former techniques allow us to cope with uncertain and incomplete knowledge whereas the latter ones lay the mathematical groundwork for a sound and complete algorithm capable to deal with regulation processes that occur at different time-scales.

The modeling framework and the simulation algorithm proposed can be applied to predict the possible qualitative behaviors of GRNs of any size and complexity, both natural and synthetic. The range of applicability of such a computational approach is quite large. First, it makes possible the validation of hypothesized models of GRNs by matching the simulated predictions against observed gene expression profiles, the derivation of the most plausible model, and the identification of parameter inequalities that account for the observations. Then, it greatly facilitates the investigation of the effects on the dynamics of a specific GRN in response to external stimuli. Finally, it allows us to build a total envisionment of the model through the generation of all possible state transitions, and to search, within them, for the parameter constraints and initial conditions that allow us to achieve a desired behavior, e.g. a stable solution. In term of tasks, besides its undoubted contribution to the comprehension of complex networks of genes and interactions, the proposed computational approach could be fruitfully used in the design of either new drugs or synthetic regulatory networks.

The code will be freely available for non-profit academic research by making a user licence request to ironi@imati.cnr.it.

## Competing interests

The authors declare that they have no competing interests.

## Authors' contributions

LI devised the work, and drafted the manuscript. Both authors participated in the design and development of the algorithms. LP implemented the software. All authors read and approved the final manuscript.
